# Supplementary figures and images for: Oocyte vitrification disrupts zygotic genome activation in embryos by impairing maternal spliceosome translation and Crxos splicing
Source: PLoS Genet. 2026 Apr 15;22(4):e1012121. doi: 10.1371/journal.pgen.1012121 (PMC13095128; doi:10.1371/journal.pgen.1012121)

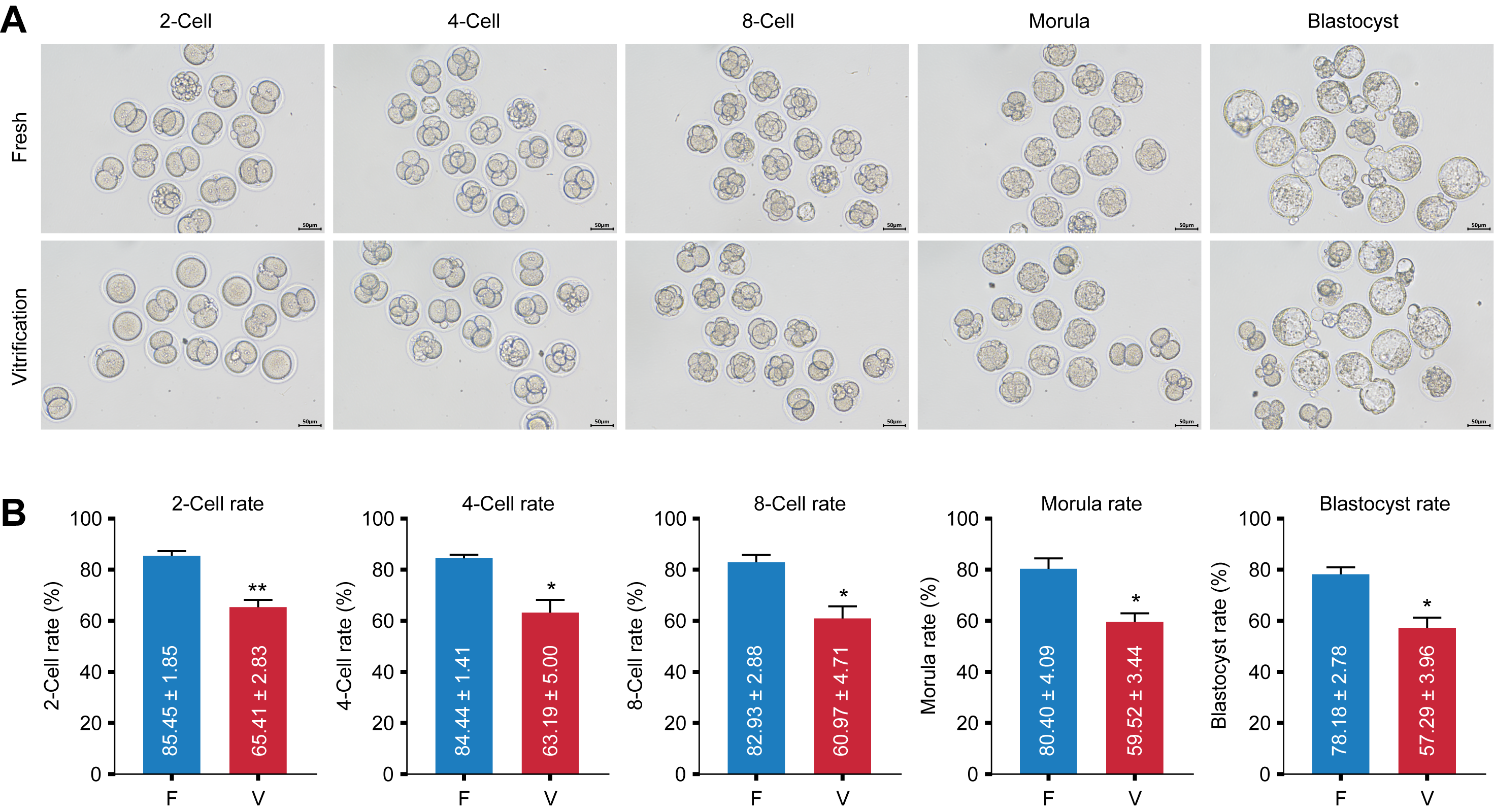

Supplement: S1 Fig — (A) Representative images of embryos at each stage post-fertilization. Scale bar = 50 µm. (B) Developmental rates of Fresh (n = 70) and Vitrification (n = 53) groups. Data are mean ± SEM from three independent experiments; *P < 0.05, **P < 0.01. (TIF) [file pgen.1012121.s001.tif]

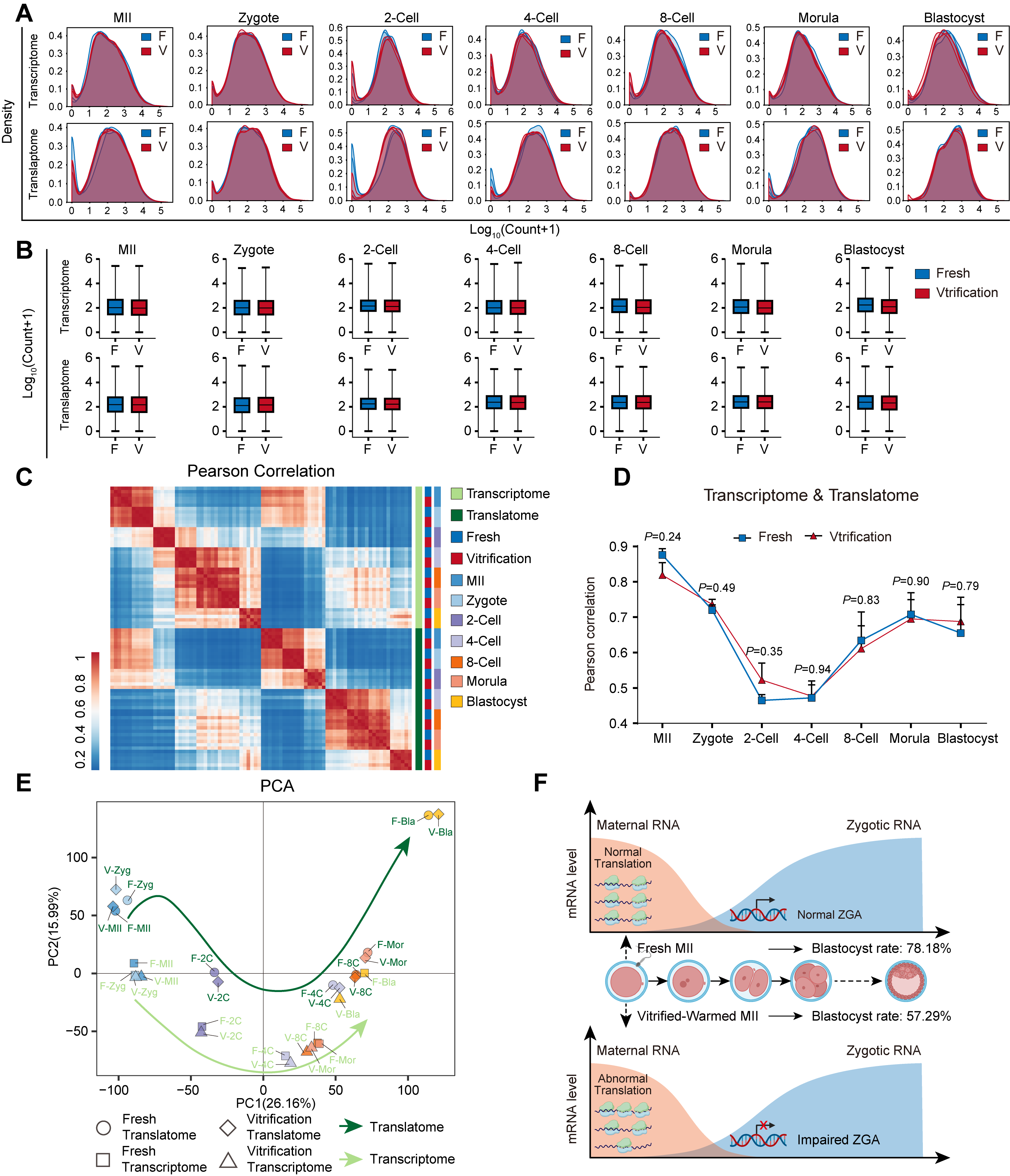

Supplement: S2 Fig — (A) Density distribution of raw counts between fresh and vitrified groups from T&T‑seq data. (B) Bar chart of total raw counts between fresh and vitrified groups from T&T‑seq data. (C) Heatmap of pearson correlation coefficients for the transcriptome and translatome. (D) Changes in transcriptome-translatome correlations induced by vitrification. (E) PCA of transcriptome and translatome. (F) Schematic summarizing the patterns of gene expression changes following vitrification. Created in BioRender. x, X. (2026) https://BioRender.com/1ned0v0. (TIF) [file pgen.1012121.s002.tif]

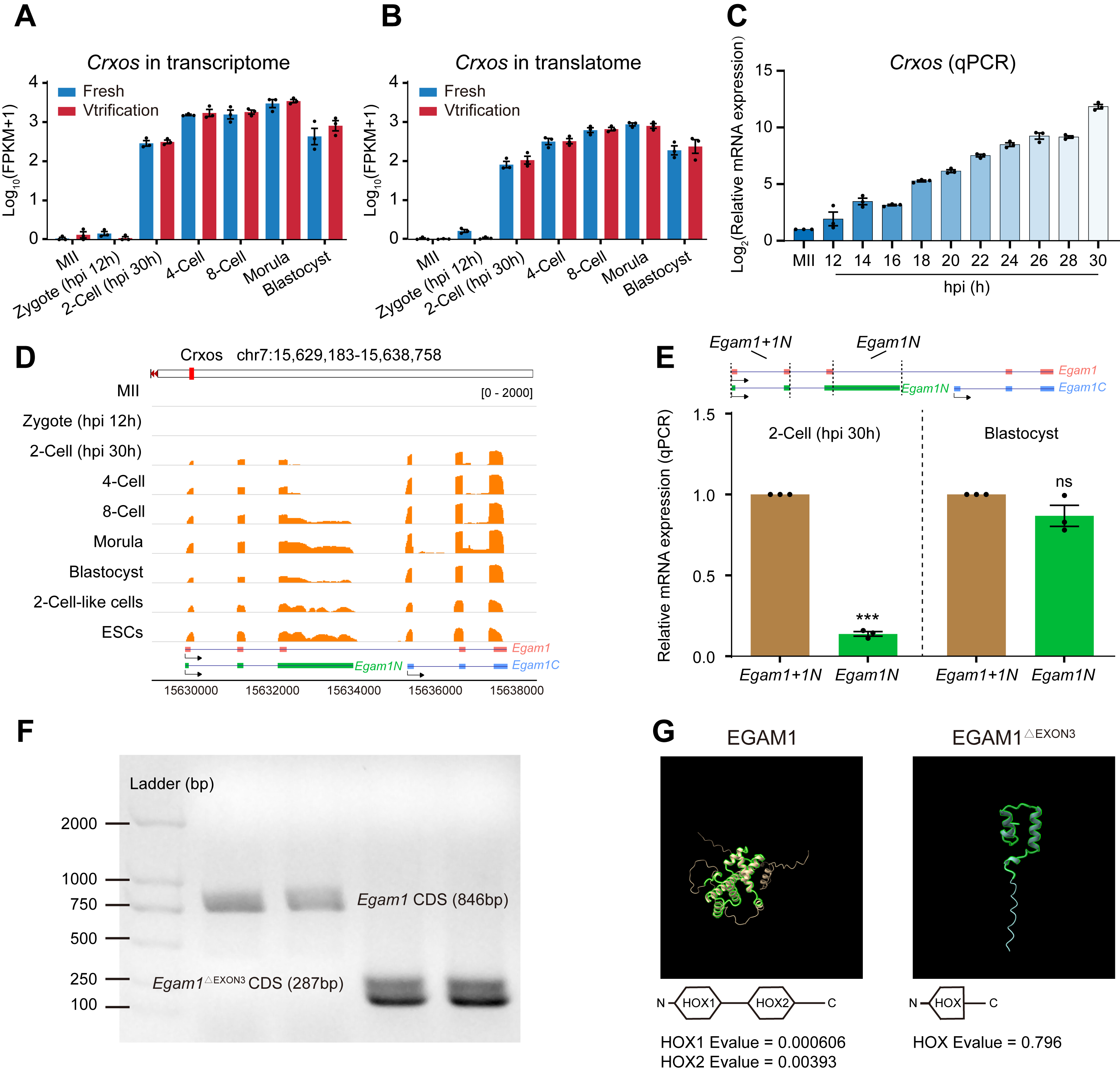

Supplement: S3 Fig — (A, B) Transcriptional and translational expression levels of Crxos across stages. (C) RT-qPCR of Crxos expression from 12 to 30 hpi (2 h intervals). (D) UCSC Browser tracks of Crxos isoforms in oocytes, embryos, 2C-like cells, and ESCs. (E) RT-qPCR of Egam1 and Egam1N transcripts in 2-cell and blastocyst stages. (F) PCR amplification of Egam1 and Egam1ΔEXON3 CDS regions. (G) Predicted protein structures of Egam1 and Egam1ΔEXON3. (TIF) [file pgen.1012121.s003.tif]

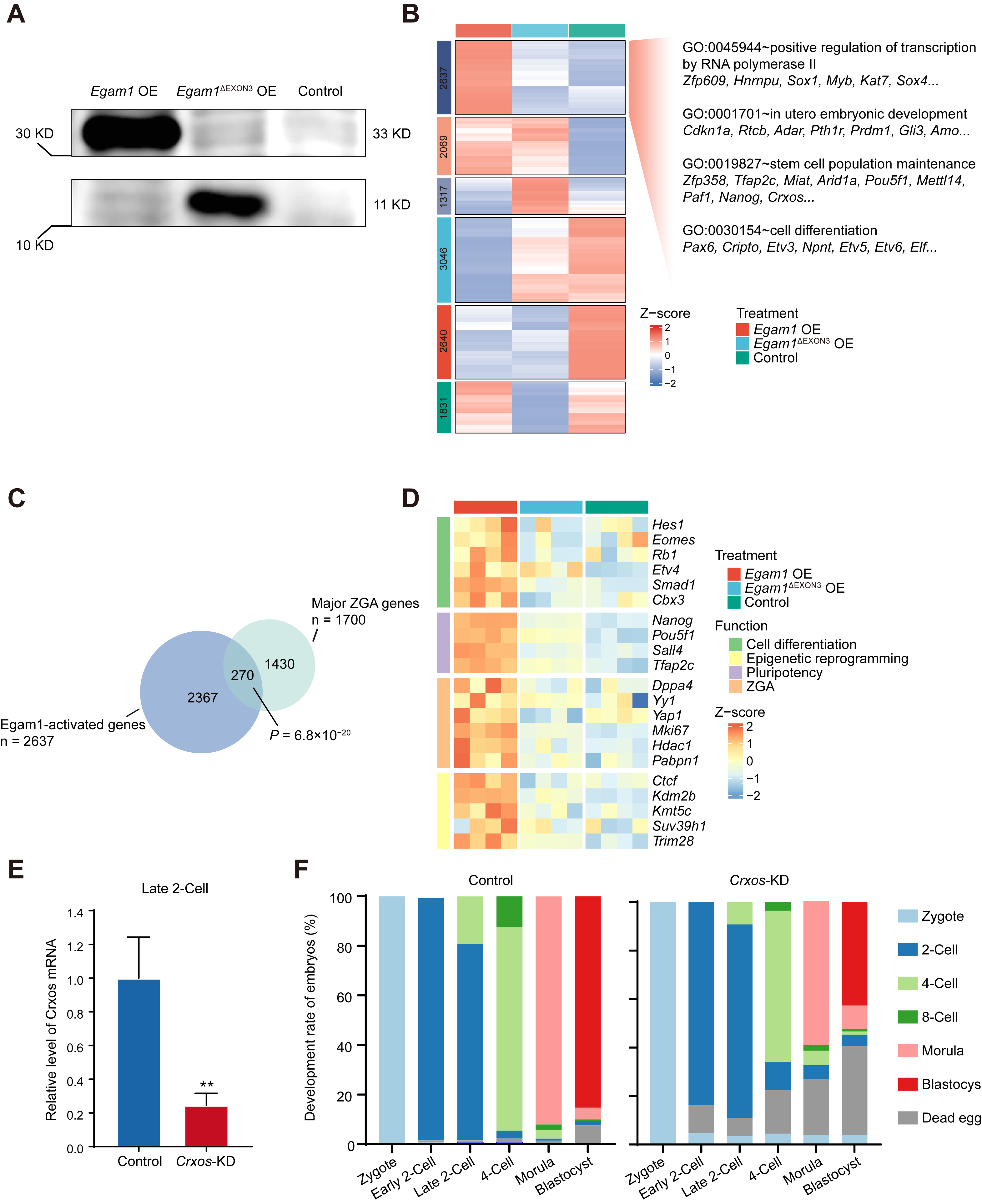

Supplement: S4 Fig — (A) Egam1 and Egam1ΔEXON3 protein expression after OE in P19 cells. (B) Transcriptional impact and GO enrichment of Egam1-activated genes. (C) Venn diagram showing the overlap between Egam1-activated genes and ZGA genes. (D) Heatmap of representative Egam1-activated genes. (E) RT-qPCR validation of Crxos-KD efficiency. (F) Stacked bar chart showing embryonic development after Crxos-KD. (TIF) [file pgen.1012121.s004.tif]

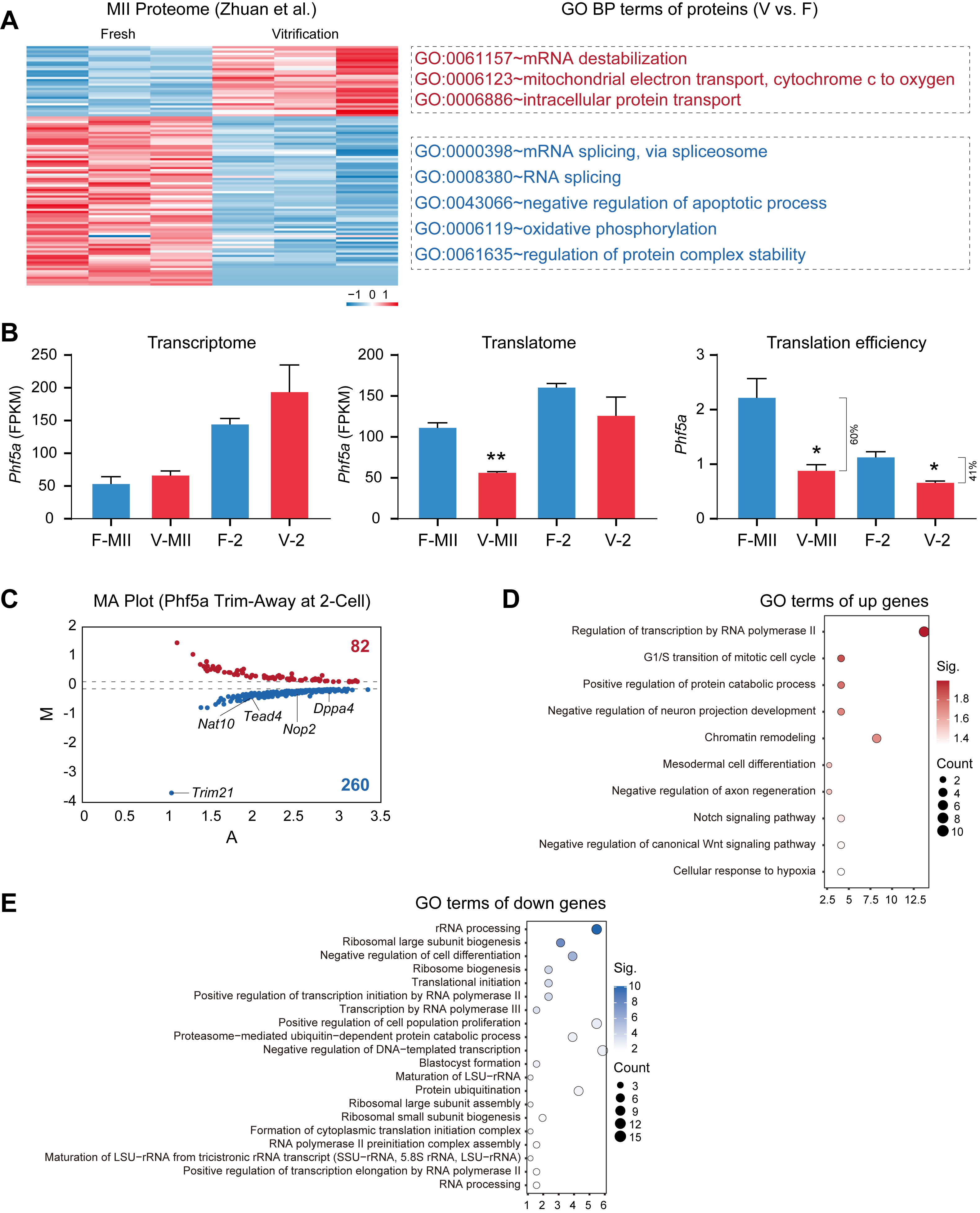

Supplement: S5 Fig — (A) GO enrichment of differentially expressed proteins (DEPs) in vitrified oocytes [44]. (B) Phf5a expression was examined by T&T-seq. Translation efficiency was calculated as the ratio of translatome to transcriptome expression levels. Data are mean ± SEM from three independent experiments; **P < 0.01, *P < 0.05. (C) MA plot showing DEGs in 2-cell embryos after Phf5a-KD. (D, E) GO enrichment of upregulated (red) and downregulated (blue) genes in Phf5a-KD embryos. (TIF) [file pgen.1012121.s005.tif]
