# Supplementary material for: Oocyte vitrification disrupts zygotic genome activation in embryos by impairing maternal spliceosome translation and Crxos splicing
Source: PLoS Genet. 2026 Apr 15;22(4):e1012121. doi: 10.1371/journal.pgen.1012121 (PMC13095128; doi:10.1371/journal.pgen.1012121)
Supplement: Attachment — Submitted filename: Response to Reviewers.docx [file pgen.1012121.s017.docx]

## **COVER LETTER**

Dear Editors,

We are pleased to submit the revised manuscript (**Manuscript ID: PGENETICS-D-26-00149**) titled "*Oocyte vitrification disrupts zygotic genome activation in embryos by impairing maternal spliceosome translation and Crxos splicing*" for potential publication in your esteemed journal, *PLOS Genetics*.

On behalf of all the authors, we extend our sincere gratitude to the editors and reviewers for their thoughtful evaluation of our work. We deeply appreciate their valuable insights and constructive feedback, which have greatly contributed to enhancing the quality of our manuscript.

In response to the reviewers’ comments and suggestions, we have thoroughly revised and optimized the manuscript. Additionally, we have provided a detailed point-by-point response to address each of the reviewers’ comments, which is appended below for your review.

We hope that our revised manuscript meets the expectations of the editorial team and reviewers and is now suitable for publication in *PLOS Genetics*. Thank you for your time and consideration, and we look forward to your decision.

Sincerely,

**Jie Yan**

Center for Reproductive Medicine, Department of Obstetrics and Gynecology, Peking University Third Hospital, Beijing 100191, China

E-mail: yanjiebjmu@bjmu.edu.cn; Telephone number: 0086-13466759981

**Guangbin Zhou**

College of Animal Science and Technology, Sichuan Agricultural University, Chengdu 611130, China.

E-mail: zguangbin@sicau.edu.cn; Telephone number: 0086-15908189189

## **Point-by-point responses to the Journal Requirements**

**Journal Requirements:**

**1.** We do not publish any copyright or trademark symbols that usually accompany proprietary names, eg ©, ®, or ™ (e.g. next to drug or reagent names). Therefore please remove all instances of trademark/copyright symbols throughout the text, including:

- ® on pages: 19, and 22.
- ™ on page: 20.

**Response:** Thank you for your valuable suggestions regarding our manuscript. We have complied with the journal requirements and removed all instances of trademark and copyright symbols throughout the text. Specifically, we have deleted:

- the ® symbols on lines 404, 405, 478 and 479
- the ™ symbols on lines 433

We have reviewed the entire manuscript to ensure that no other trademark or copyright symbols remain.

**2.** We notice that your supplementary figures are uploaded with the file type 'Figure'. Please amend the file type to 'Supporting Information'. Please ensure that each Supporting Information file has a legend listed in the manuscript after the references list.

**Response:** Thank you. We have amended the file type of our supplementary figures from 'Figure' to 'Supporting Information' as requested. Additionally, we have ensured that each Supporting Information file now includes a corresponding legend, which has been listed in the manuscript after the references list.

**3.** Some material included in your submission may be copyrighted. According to PLOSu2019s copyright policy, authors who use figures or other material (e.g., graphics, clipart, maps) from another author or copyright holder must demonstrate or obtain permission to publish this material under the Creative Commons Attribution 4.0 International (CC BY 4.0) License used by PLOS journals. Please closely review the details of PLOSu2019s copyright requirements here: PLOS Licenses and Copyright. If you need to request permissions from a copyright holder, you may use PLOS's Copyright Content Permission form.

Please respond directly to this email and provide any known details concerning your material's license terms and permissions required for reuse, even if you have not yet obtained copyright permissions or are unsure of your material's copyright compatibility. Once you have responded and addressed all other outstanding technical requirements, you may resubmit your manuscript within Editorial Manager.

Potential Copyright Issues:

i) Figures 1B, 5G, 7, and S2D. Please confirm whether you drew the images / clip-art within the figure panels by hand. If you did not draw the images, please provide (a) a link to the source of the images or icons and their license / terms of use; or (b) written permission from the copyright holder to publish the images or icons under our CC BY 4.0 license. Alternatively, you may replace the images with open source alternatives. See these open source resources you may use to replace images / clip-art:

- https://commons.wikimedia.org
- https://openclipart.org/

Note: If the figure is created through BioRender, please confirm that you hold a Premium account and provide a pdf copy of the CC BY 4.0 Licence as provided by BioRender. For instructions on how to generate a CC BY 4.0 license for your figure, please see the guidelines here: https://help.biorender.com/hc/en-gb/articles/21282341238045-Publishing-in-open-access-resources.

If you are using the free assets from BioRender, we are unable to publish these images as they are licenced under a stricter licence than CC BY 4.0. In this case we ask you to remove the BioRender images and replace them with open source alternatives.

See these open source resources you may use to replace images / clip-art:

- https://bioart.niaid.nih.gov/
- https://bioicons.com/
- https://healthicons.org/
- https://scidraw.io/
- https://reactome.org/icon-lib
- https://www.phylopic.org/images
- https://journals.plos.org/plosbiology/article?id=10.1371/journal.pbio.3002395

**Response:** Thank you. We have carefully reviewed the potential copyright issues and are pleased to provide the requested information. Regarding Figures 1B, 5G, 6F, 7, and S2F: We confirm that all these figures were created using BioRender. The corresponding author holds a Premium BioRender account, which allows us to generate figures under the Creative Commons Attribution 4.0 International (CC BY 4.0) license as required by PLOS. We have attached a PDF copy of the CC BY 4.0 license for each of the mentioned figures, as provided by BioRender. These licenses confirm that the figures may be published under the CC BY 4.0 license.

**4.** Please amend your detailed Financial Disclosure statement. This is published with the article. It must therefore be completed in full sentences and contain the exact wording you wish to be published.

(1) State the initials, alongside each funding source, of each author to receive each grant. For example: "This work was supported by the National Institutes of Health (####### to AM; ###### to CJ) and the National Science Foundation (###### to AM)."

(2) State what role the funders took in the study. If the funders had no role in your study, please state: "The funders had no role in study design, data collection and analysis, decision to publish, or preparation of the manuscript".

**Response:** Thank you. We have revised it according to your instructions to ensure it meets the journal's requirements. We have amended the Financial Disclosure statement as follows:

Funding sources：This work was supported by the National Key Research and Development Program of China (2021YFD1200403 to GZ, 2022YFC2703000 to JY) and the National Natural Science Foundation of China Major Program (T2293764 to JY).

Role of funders: The funders had no role in study design, data collection and analysis, decision to publish, or preparation of the manuscript. We confirm that the statement is now written in complete sentences and reflects the exact wording we wish to be published.

**Note:** If the reviewer comments include a recommendation to cite specific previously published works, please review and evaluate these publications to determine whether they are relevant and should be cited. There is no requirement to cite these works unless the editor has indicated otherwise.

**Response:** Thank you. We have carefully reviewed the reviewer comments and confirm that no recommendations to cite any specific previously published works were included.

## **Point-by-point responses to the Reviewers' Comments**

### **Reviewer 1:**

**Overall Impressions**

This study provides a compelling mechanistic link between oocyte vitrification and compromised embryonic development through integrated transcriptome and translatome analyses in mouse oocytes. The authors demonstrate that vitrification specifically disrupts maternal mRNA translation—without affecting global transcriptional output—leading to suppressed expression of spliceosome components, including Phf5a. This perturbation results in persistent alternative splicing defects in 2-cell embryos, notably affecting the zygotic genome activation (ZGA) regulator *Crxos* (*Egam1*), where the functional full-length transcript is depleted while a truncated non-coding variant is elevated. Functional assays confirm that *Crxos* loss in 2-cell embryos impairs both developmental progression and global transcriptional activity, likely through defective RNA Pol II recruitment and elongation at ZGA genes. These findings not only advance our fundamental understanding of post-vitrification embryonic defects but also carry significant translational implications for optimizing cryopreservation protocols in reproductive medicine. The data are robust and well-supported, and the manuscript warrants acceptance after minor revisions.

**Response:** We sincerely thank Reviewer #1 for their constructive comments and compliments on our manuscript. We have carefully addressed each suggestion and incorporated the necessary revisions into the manuscript. The presentation of the content has been significantly improved in line with these valuable recommendations.

**General Suggestions**

**1.** Please carefully review the manuscript for grammatical issues and improve the clarity of the language throughout the text.

**Response:** Thank you for your valuable input on improving the manuscript's language. We have carefully reviewed the entire text for grammatical issues and language clarity, and have made comprehensive revisions accordingly. In addition to incorporating the minor suggestions and grammar corrections, we have also made further improvements at multiple locations, including lines 65-66, 71, 88-89, 129, 132, 138, 144-145, 233, 237, 239, 244, 247, 264, 269, 282, 309, 311, 314, 344, 345, 390, 391, 398, 407-408, 522, 534, 859, 874, 912, 918, 933, 951-952, 954-955, 959, and 967-968, to enhance the overall readability.

**2.** Please ensure that all gene names are italicized consistently throughout the manuscript.

**Response:** Thank you for drawing our attention to the gene name formatting. We have thoroughly reviewed the entire manuscript and ensured that all gene names are italicized consistently throughout the text, including in the main text, figures, figure legends, and supplementary materials. For example, gene names such as *Crxos*, *Egam1*, and *Phf5a* are now correctly formatted in italics, while protein names remain in regular type.

**3.** Please ensure that all figure legends are comprehensive yet concisely presented.

**Response:** Thank you for your helpful guidance on improving the figure legends. We have thoroughly reviewed all figure legends to ensure they are both comprehensive and concisely presented. Specifically, we have: (1) Verified that each figure legend clearly describes the experimental design, sample sizes, and statistical methods. (2) Eliminated redundant or overly detailed descriptions while retaining all essential information needed for the figure to be interpreted independently of the main text. (3) Ensured consistent formatting and terminology across all legends.

**Minor Suggestions and Grammar Corrections**

**Author Summary**

**Line 48-50:** The sentence structure is slightly awkward. Consider revising to: "Our findings provide a mechanistic basis for optimizing cryopreservation protocols in reproductive medicine."

**Response:** Thank you. This has been corrected in the revised manuscript (Lines 48-49).

**Introduction**

**Line 53:** The word "dominant" is somewhat subjective and imprecise. Please replace with "a standard" to more objectively reflect its clinical status.

**Response:** Thank you. This has been corrected (Line 52).

**Line 58:** Please revise "This developmental compromise poses a significant barrier..." to "This diminished developmental potential (e.g., reduced blastocyst rates) poses a significant barrier..." for greater specificity and impact.

**Response:** Thank you. This has been revised in the manuscript (Lines 57-58).

**Line 77:** Please replace "maternal mRNA" with "maternal mRNAs".

**Response:** Thank you. These corrections have been made (Line 78).

**Line 93:** Please replace "spliceosome associated mRNAs" with "spliceosome-associated mRNAs".

**Response:** Thank you. This has been corrected (Line 93).

**Line 94:** Please replace "failed transcriptional activation" with "the failure of transcriptional activation".

**Response:** Thank you. The model has been included (Line 94).

**Line 101:** Please replace "seek" with "sought".

**Response:** Thank you. This has been corrected (Line 101).

**Results**

**Line 117:** Please replace "before subsequently increasing" with "and subsequently increased".

**Response:** Thank you. This has been revised in the manuscript (Line 122).

**Line 118:** Please replace "fresh group" with "the fresh group".

**Response:** Thank you. This has been revised (Lines 124-125).

**Line 150:** Please replace "subsequent developmental" with "subsequent development".

**Response:** Thank you. This has been corrected (Line 157).

**Line 157:** Please replace "stress responsive processes" with "stress-responsive processes".

**Response:** Thank you. These corrections have been made (Lines 164).

**Line 169-170:** Please replace "with skipped exon being the predominant form" with "with skipped exons being the predominant form".

**Response:** Thank you. This has been revised in the manuscript (Lines 176-177).

**Line 174:** Remove the comma before "and".

**Response:** Thank you. This has been corrected (Line 181).

**Line 183-184:** Please replace "*Crxos*'s full-length transcript" with "the full-length transcript of *Crxos*".

**Response:** Thank you. This has been corrected (Lines 190-191).

**Line 185:** Please replace "as further validated by PCR" with "as validated by PCR".

**Response:** Thank you. These corrections have been made (Line 192).

**Line 196:** Please replace "stress related pathways" with "stress-related pathways".

**Response:** Thank you. This has been corrected (Lines 202-203).

**Line 200:** Please replace "effectively reduced" with "was found to effectively reduce".

**Response:** Thank you. This has been revised in the manuscript (Line 207).

**Line 202:** Please replace "decreased rates of 2 cell to 4 cell transition" with "decreased rates of transition from 2-cell to 4-cell".

**Response:** Thank you. This has been corrected (Line 209).

**Line 210-211:** Please replace "genome wide RNA Pol II binding profiles" with "genome-wide RNA Pol II binding profiles".

**Response:** Thank you. This has been corrected (Lines 218-219).

**Line 212:** Please replace "well defined Pol II peaks" with "well-defined Pol II peaks".

**Response:** Thank you. This has been corrected (Line 220).

**Line 245:** Please replace "enriched among" with "enriched in".

**Response:** Thank you. This has been corrected (Line 253).

**Line 269-270:** Please replace "reduction of maternal spliceosome protein directly disrupts alternative splicing" with "reduction of the maternal spliceosome protein Phf5a directly disrupts alternative splicing".

**Response:** Thank you. This has been revised in the manuscript (Lines 279-280).

**Discussion**

**Line 286-287:** Please replace "a large number of aberrant alternative splicing genes" with "a large number of genes exhibiting aberrant alternative splicing".

**Response:** Thank you. This has been corrected (Lines 296-297).

**Line 310:** Please replace "genome wide mechanism" with "genome-wide mechanism".

**Response:** Thank you. This has been corrected (Line 320).

**Materials and Methods**

**Line 354-355:** Please replace "oocytes were harvested 14 h post-hCG injection" with "oocytes were harvested at 14 h post-hCG injection".

**Response:** Thank you. This has been corrected (Lines 364-365).

**Line 371:** Please replace "37.5°C heated stage" with "37.5°C-heated stage".

**Response:** Thank you. This has been corrected (Line 381).

### **Reviewer 2:**

In this manuscript, Qin et al. describe analyses of the effects of oocyte vitrification on gene expression prior to and following zygotic genome activation, to try to identify pathological effects that could contribute to reported reduced IVF success resulting from the vitrification process. They note that indeed oocyte vitrification reduces the rate of developmental progression to blastocyst stage, consistent with prior reports. The authors perform transcriptomic and translatomic measurements to identify potential changes to the gene expression machinery. These experiments can be challenging to interpret if there are global effects, and in fact the raw numbers of differentially expressed genes (DEGs) initially appear to be quite small considering the thousands of genes expressed prior to and post-ZGA. However, the authors do identify some important differentially translated genes such as *Phf5a*, an important splicing factor, and provide some compelling analyses that suggest a causal role for vitrification in embryonic splicing-associated defects. The overall research question is important given the lack of knowledge of manipulations such as vitrification on oocyte and early embryonic gene expression, and the authors provide important data on potential gene expression defects with important new genomic datasets that may provide quite useful to the field that I believe make this story worthy of publication in *PLOS Genetics*.

**Response:** We sincerely thank Reviewer #2 for the positive and encouraging comments on our manuscript. We are pleased that the reviewer found our study to be important and worthy of publication in *PLOS Genetics*. We have carefully considered all the reviewer's comments and revised the manuscript accordingly. We believe these revisions have further strengthened the study.

**Major points:**

**1.** “…the vitrification group showed higher transcriptome-translatome correlation in MII oocytes and 8-cell embryos but lower correlation in at the 2-cell and blastocyst stages (S2B Fig.)” The differences look very subtle in this figure, given a lack of statistical analysis controlling for multiple testing across various timepoint comparisons it is not apparent if this is a biologically meaningful result.

**Response:** Thank you for the valuable comments regarding the interpretation of the original S2B Fig. We agree that the differences in transcriptome–translatome correlations between the fresh and vitrification groups across developmental stages appeared subtle in the original S2B Fig (**now revised S2D Fig**). To address this, we first corrected a previous error in which the order of the fresh and vitrification groups had been inadvertently swapped in the correlation analysis. We then performed two‑tailed *t*‑tests for each stage followed by Benjamini–Hochberg correction for multiple testing. No statistically significant differences were observed at any stage (*P* > 0.05). Although subtle trends were observed at certain stages, the effect sizes were modest; we have therefore moderated the corresponding description in the revised manuscript to avoid overinterpretation (**revised manuscript lines 122-125**). Given the limited sensitivity of the global correlation metric to stage‑specific regulatory changes, this analysis is presented only as supportive evidence for the effect of oocyte vitrification on global transcriptome–translatome correlation, and the core conclusions of our study do not rely on it.

**2.** If translation utilizing maternally supplied ribosomes/mRNAs was disrupted with consequential effects on ZGA why were there no DEG’s in transcriptome comparisons in post-ZGA stages (8-cell/morula/blastocyst)? It would be helpful to add percentages of genes that were unaffected vs. differential - e.g. out of 12,000 genes detected at the level of transcript abundance and ribosome-association, 1.8% or 218 were significantly differentially expressed. Even at the time points with the most DEGs, it would appear that the vast majority of genes were identically expressed between fresh vs. vitrified samples. Why would the effects of vitrification lead to alterations in such a small subset of transcripts? It would seem to me that the fact that the vast majority of genes were not differentially expressed at the level of transcript abundance/translation would support the null hypothesis that the gene expression apparatus of the oocyte/early embryo is largely unaffected by vitrification? Or are only a small fraction of highly expressed genes detected in these analyses, potentially leading to an underestimation of the effects on gene expression?

**Response:** Thank you for raising these important points. We address them as follows.

Regarding the absence of DEGs in transcriptome comparisons at post‑ZGA stages (8‑cell, morula, and blastocyst), we propose the following explanation. Based on the developmental phenotypes of embryos derived from vitrified‑warmed oocytes, the reduced blastocyst rate primarily resulted from 2‑cell arrest (**Fig 1A**), which coincides with the ZGA window. Thus, although the collected 2‑cell embryos appeared morphologically normal, some had already undergone aberrant ZGA and were destined to arrest. Embryos that developed to the 4‑cell stage had passed the arrest point, but a subset retained transcriptional abnormalities due to prior ZGA defects. In contrast, morphologically normal 8‑cell embryos, morulae, and blastocysts had recovered from vitrification‑induced damage and successfully passed through the ZGA window, after which their subsequent development relies entirely on the embryonic transcriptome. Under our sampling conditions, DEGs were detected during ZGA, whereas transcriptome profiles converged at post‑ZGA stages. Therefore, the most vulnerable window of early embryonic development following oocyte vitrification is the ZGA period (2‑ to 4‑cell stage), which was the primary focus of our study.

Regarding the concern that the small number of DEGs might support the null hypothesis that vitrification has little effect on the gene expression machinery, we offer the following analysis. First, at the MII stage, no DEGs were detected in the transcriptome, whereas the translatome showed substantial changes (105 upregulated, 113 downregulated). This indicates that vitrification primarily perturbs maternal mRNA translation, consistent with the transcriptionally silent and translation‑dependent regulatory landscape of oocytes. Second, in the integrated analysis of transcriptome and translatome, we excluded non‑coding RNAs and retained only genes with raw counts with a sum >10 across three biological replicates for differential expression analysis to ensure data robustness. This filtering removed several differentially expressed non‑coding RNAs, such as the non‑coding repeat element MERVL‑int, used as a ZGA marker in our study (**Fig 2D**). Therefore, although the majority of genes were not significantly affected, it remains possible that dysregulation of a few key genes, including *Phf5a* (**Fig 6**), contributes to the early developmental arrest observed in this study.

**3.** Any sense whether global levels of translation were affected? Spike-in or other controls for global effects on gene expression can frequently unmask deficits in translation or RNA stability occurring across the transcriptome that may be otherwise obscured by common normalizations used in genomic analyses (e.g. TPM normalization). How would the analyses changed if accounting for bulk changes in genome-wide transcription as described in Fig. 2E?

**Response:** Thank you for your insightful comments regarding global translation levels, the necessity of spike‑in controls, and the potential impact of reduced global transcriptional activity on our analyses. We address these points as follows:

In response to the reviewer’s question on whether global translation levels were affected, we calculated global translation efficiency (translatome/transcriptome) and found that it was significantly higher in 2‑cell embryos derived from vitrified oocytes compared to fresh controls (**revised Fig. 1E and 1F; revised manuscript lines 141-142**).

Regarding the concern that spike‑in controls can unmask global defects that may be obscured by conventional normalization, we agree with the reviewer that spike‑in provides the most rigorous approach for evaluating global effects, particularly when absolute quantification is required. Given that the present study primarily used T&T‑seq to compare relative expression differences between fresh and vitrification groups, spike‑in controls were not included in the library preparation process. To minimize technical variability, we ensured identical cell numbers (n=10), amplification cycles, and library concentrations across groups, consistent with previous reports (**PMID: 36042231, 40680131**). To further rule out the possibility that conventional normalization might mask global defects in gene expression, we analyzed the distribution of raw counts and observed no evident global shifts (**revised S2A Fig; revised manuscript lines 114-118**). Thus, relative quantification in this study appears reliable, although absolute quantification would require spike‑in correction.

Regarding whether the genome‑wide reduction in transcriptional activity shown in Fig. 2E could affect our analyses, we note that EU incorporation measures the rate of nascent RNA synthesis, whereas RNA‑seq quantifies total RNA content. We further compared the total raw counts between fresh and vitrification groups and found comparable levels with no significant difference (**revised S2B Fig; revised manuscript lines 114-118**). Moreover, the RNA detected by transcriptome analysis in 2‑cell embryos includes not only nascent embryonic RNA but also maternally stored RNA (**PMID: 39823229, 31777931, and 41755639**). Therefore, the overall reduction in transcriptional activity reflected by EU results does not represent the overall trend of the transcriptome (**PMID: 41015043**).

Based on the above analyses, the conclusions drawn from the relative quantification framework in this study reliably reflect the biological differences between the fresh and vitrification groups.

**4.** Supplementary tables should be provided with a list of the specific DEG’s described in key figures, such as Fig. 2A.

**Response:** Thank you for your valuable suggestions regarding supplementary tables. In response, we have prepared five supplementary tables listing the genes and alternative splicing events presented in key figures, which we believe will enhance the transparency and usability of our data. Specifically:

S1 Table provides the complete T&T‑seq dataset from vitrified‑warmed MII oocytes and their derived preimplantation embryos, as shown in Fig. 1B.

S2 Table provides the list of major ZGA genes identified in Fig. 2A for both the fresh and vitrification groups.

S3 Table provides the list of differential alternative splicing events during oocyte and early embryonic development from T&T‑seq data, corresponding to Fig. 3A.

S4 Table provides the list of differential alternative splicing events between the fresh and vitrification groups during ZGA, corresponding to Fig. 3B.

S5 Table provides the list of differential alternative splicing events upon Phf5a Trim‑Away at the 2‑cell stage, corresponding to Fig. 6K.

These supplementary data files have been uploaded as “Supporting Information” and are now listed after the references section (**Lines 996-1004**), as required.

**5.** In general there are issues with low figure resolution. Figures should be exported at a much higher resolution. For example, Fig. 4C and 4D, 5B etc. are very difficult to read.

**Response:** Thank you for your constructive feedback regarding figure resolution. We have carefully addressed this issue and revised the figures accordingly. To address this, we have thoroughly reviewed and regenerated all figures at an appropriately high resolution, with particular attention to the figures mentioned (including Fig. 4C, 4D, and 5B), which are now clearly legible.

**6.** Authors should quantify the RT-PCR experiment shown in Fig. 4B with a statistical analysis of the spliced vs. unspliced form from analyses of the three replicates.

**Response:** Thank you for your valuable suggestion to quantify the RT-PCR results shown in Fig. 4B. In response, we have performed statistical analysis of the *Egam1* (full-length, 452 bp) and *Egam1*^ΔEXON3^ (exon 3 skipping, 279 bp) bands from three independent replicate experiments. As described in previous reports (**PMID: 26721495**), the frequency of exon 3 skipping was calculated by dividing the pixel intensity of the exon 3 skipping band by the combined pixel intensity of both the full-length and exon 3 skipping bands. For each replicate, 10 embryos at 2-cell stage were collected from both the fresh and vitrification groups, and PCR amplification was performed under identical cycle numbers. Images from all three biological replicates were obtained from the same gel to ensure consistency. Statistical analysis confirms that the frequency of exon 3 skipping in *Egam1* is significantly increased in 2-cell embryos derived from vitrified oocytes (*P* < 0.05), which is consistent with the T&T-seq data shown in Fig. 4A. These quantified results have been incorporated into the revised manuscript as Fig. 4C.

The quantification method has been added to the Materials and Methods section 4.7 (**Lines 427-432**), and the corresponding figure legend has been updated in Fig. 4 to include panel C (**Lines 901-904**). The revised manuscript text now cites Fig. 4C alongside Fig. 4A and 4B to support the finding that exon 3 skipping frequency is elevated in the vitrification group (**Line 192**).

**7.** To help interpret the results it would be helpful to be able to compare the decrease for proteins such as Phf5a as measured by T&T-seq vs. the Western blotting by providing additional numbers, e.g. for each protein measured how much was it decreased in this dataset at the levels of translation vs. transcript abundance vs. the net effects on protein levels? This would also help disentangle changes to protein stability vs. synthesis.

**Response:** Thank you for your valuable suggestion to compare the decrease in Phf5a protein as measured by T&T-seq with western blotting, and to delineate the changes in protein stability and protein synthesis. To facilitate interpretation, we compared *Phf5a* at the levels of transcription, translation, and translation efficiency. At the transcriptional level, no significant differences were observed between fresh and vitrification groups in either MII oocytes or 2‑cell embryos. At the translational level, *Phf5a* was significantly reduced only in vitrified MII oocytes, but not in 2‑cell embryos. Given that the rate of protein synthesis depends on the translation efficiency of mRNA (**PMID: 41038299**), we analyzed translation efficiency of *Phf5a* in both stages. In MII oocytes, translation efficiency of *Phf5a* decreased by 60% in the vitrification group, consistent with the 40% reduction in protein levels by western blotting. In 2‑cell embryos, translation efficiency decreased by 41%, while western blotting showed a 60% reduction. These results indicate that vitrification reduces Phf5a protein expression primarily by impairing its translation efficiency. The translation efficiency data have been added as S5B Fig, with the corresponding legend in S5 Fig (**Lines 990-993**), and are cited accordingly in the Results section (**Line 260**).

**8.** How much knockdown of Phf5a was achieved? It looks like the knockdown was quite subtle in Fig. 6H – is it known if this gene is dosage sensitive where a small degree of knockdown would be predicted to be biologically significant?

**Response:** Thank you for your insightful question regarding the efficiency of Phf5a-KD and its potential dosage sensitivity. We quantified Phf5a-KD efficiency using the Trim-Away approach. As shown in Fig. 6H, Phf5a protein levels were reduced by approximately 16% in the KD group compared to the control. Although this reduction appears modest, it led to significant biological consequences: the rate of 2‑cell to 4‑cell transition decreased from 88.89 ± 3.87% to 65.28 ± 5.01%, and blastocyst formation decreased from 84.72 ± 3.47% to 61.11 ± 1.39%. At the molecular level, this moderate KD resulted in 260 downregulated genes enriched in transcriptional regulation and blastocyst development, along with 1,072 differential alternative splicing events, which primarily affected genes involved in cell cycle and embryonic development. Indeed, the critical role of Phf5a in cellular function is supported by evidence that its knockdown impairs self‑renewal in mouse ESCs and promotes apoptosis in melanoma cells (**PMID: 27749823, 39212334**). These findings indicate that Phf5a is dosage‑sensitive, as even a 16% reduction in protein levels is sufficient to perturb both downstream gene expression and preimplantation development.
